# Masses of developmental and genetic origin affecting the paediatric craniofacial skeleton

**DOI:** 10.1007/s13244-018-0623-4

**Published:** 2018-05-15

**Authors:** Salvatore Stefanelli, Pravin Mundada, Anne-Laure Rougemont, Vincent Lenoir, Paolo Scolozzi, Laura Merlini, Minerva Becker

**Affiliations:** 1 Division of Radiology, Department of Imaging and Medical Informatics, Geneva University Hospitals, Rue Gabrielle-Perret-Gentil 4, 1211 Geneva 14, Switzerland; 2 Division of Clinical Pathology, Department of Genetic and Laboratory Medicine, Geneva University Hospitals, Rue Gabrielle-Perret-Gentil 4, 1211 Geneva 14, Switzerland; 3 Clinic of Maxillo-facial Surgery, Department of Surgery, Geneva University Hospitals, Rue Gabrielle-Perret-Gentil 4, 1211 Geneva 14, Switzerland

**Keywords:** Head and neck, Maxillofacial, Developmental lesions, CT, MRI

## Abstract

**Abstract:**

Although rare, masses and mass-like lesions of developmental and genetic origin may affect the paediatric craniofacial skeleton. They represent a major challenge in clinical practice because they can lead to functional impairment, facial deformation and disfigurement. The most common lesions include fibrous dysplasia, dermoid cysts, vascular malformations and plexiform neurofibromas. Less common lesions include torus mandibularis and torus palatinus, cherubism, nevoid basal cell carcinoma syndrome, meningoencephalocele and nasal sinus tract. This article provides a comprehensive approach for the evaluation of children with masses or mass-like lesions of developmental and genetic origin affecting the craniofacial skeleton. Typical findings are illustrated and the respective roles of computed tomography (CT), cone beam CT (CBCT), magnetic resonance imaging (MRI) with diffusion-weighted imaging (DWI) sequences and ultrasonography (US) are discussed for the pre-therapeutic assessment, complex treatment planning and post-treatment surveillance. Key imaging findings and characteristic clinical manifestations are reviewed. Pitfalls of image interpretation are addressed and how to avoid them.

**Teaching points:**

*• Masses of developmental and genetic origin may severely impair the craniofacial skeleton.*

*• Although rare, these lesions have characteristic imaging features.*

*• CT, MRI and ultrasonography play a key role in their work-up.*

*• Recognition of pivotal imaging pearls and diagnostic pitfalls avoids interpretation errors.*

## Introduction

Masses and mass-like lesions related to various developmental and genetic conditions can affect the developing craniofacial skeleton. The majority of masses and mass-like conditions of developmental/genetic origin are benign. Some of these conditions, such as torus palatinus and torus mandibularis, require no treatment other than alleviation of parental anxiety. Other conditions affecting the developing craniofacial skeleton, such as fibrous dysplasia, ossifying fibroma, familial gigantiform cementoma, cemento-osseous dysplasia, hereditary multiple osteochondroma or plexiform neurofibroma, may cause functional impairments due to the proximity to important neurovascular structures, organs of special senses and developing dentition. Cosmetic deformities, due to the lesion itself or due to treatment-related asymmetric facial growth, may cause a significant psychosocial impact on the patient’s life. Management of these patients, therefore, often requires close interdisciplinary work-up, precise and often complex treatment planning strategies and post-treatment surveillance into adulthood. Radiologists, as part of an interdisciplinary team, play an important role in the management of these young patients. In addition, as these rare lesions may mimic neoplasms of the craniofacial skeleton and vice versa, it is imperative to be aware of their characteristic imaging features in order to avoid unnecessary biopsy and expensive follow-up examinations.

To the best of our knowledge, a review of the imaging features of these rare masses and their impact on treatment has not been published in the English literature during the past 20 years. Most published articles on the subject are isolated case reports or small case series dealing with the clinical presentation and with patient management. This article attempts to provide a comprehensive radiological review of the most common developmental masses involving the craniofacial skeleton, along with their multimodality imaging features, clinical manifestations and the role of imaging in their pluridisciplinary management [1–5].

## Imaging techniques

The majority of masses and mass-like conditions of developmental/genetic origin are benign. Imaging narrows down the differential diagnosis and helps in planning patient management. Traditionally, ultrasonography (US) and conventional x-ray radiography have been the mainstays of imaging in paediatric lesions. US allows differentiation between solid and cystic lesions of the facial soft tissues and enables rapid assessment of the vascularisation and localisation of the extraosseous components. The US transducer types to be used should be adapted to the small parts investigated. High-frequency linear array transducers (> 8 MHz, often > 10 MHz) yield excellent spatial resolution, but at the expense of a shallower depth of penetration. US should be ideally coupled with a Doppler evaluation. Based on the Doppler flow waveform, differentiation between infantile haemangioma, a vascular tumour gradually involuting over the years, from low-flow (venous), lymphatic or high-flow (arteriovenous) vascular malformations is facilitated. However, US has a very limited role in the evaluation of the craniofacial skeleton itself. Imaging techniques using ionising radiation, including orthopantomography (OPT), computed tomography (CT) and cone beam CT (CBCT) warrant careful use in accordance with the ALARA (As Low As Reasonably Achievable) principle [6]. As OPT is associated with a modest radiation exposure and gives a reasonable overview of the facial skeleton, gross lesion features (osteolytic, osteosclerotic vs. mixed pattern), gross lesion margins (well-delineated vs. poorly delineated, expansile vs. non-expansile, effect on teeth structures, condyle or inferior alveolar canal), lesion location and overall effect on the skeletal growth (asymmetry and deformity) can already give important clues for the differential diagnosis. According to the size and age of the patient, a wide range of preset parameters (kV and mA) can be chosen according to vendor specifications.

After the initial evaluation, the first decision is whether to use CT or magnetic resonance imaging (MRI) for further work-up or whether the acquired US and OPT images enable reasonable patient management without further imaging. If the lesion is suspected to be of vascular nature, to have a large extraosseous component or of the type that may have a small intracranial connection (e.g. dermoid cyst), MRI is the modality of choice. Many times, however, the exact nature of the lesion is not known and the clinical presentation suggests a primary intraosseous lesion, in which case, CT becomes the imaging modality of choice. Nevertheless, it is important to note that, in cases with involvement of the skull base or prior to planning complex surgery, both MRI and CT/CBCT are required. High-resolution MRI, low-dose CT and CBCT are the standard of care for the assessment of facial masses with skeletal involvement or of primary craniofacial skeletal lesions. When performing CT or CBCT, the radiation dose is usually reduced as much as possible by limiting the scanned area to the absolute minimum, by avoiding the lens if possible and by applying a low-dose protocol [6]. In general, CBCT gives a 2–5 times lower radiation exposure than CT, which makes it a preferred imaging modality. Although CBCT provides superb resolution of the bony and dental structures, it has poor soft tissue resolution. Its long acquisition time (up to 20s for one rotation) limits the use in young children, where motion artefacts due to limited cooperation are common. For CT and CBCT, 0.6–1-mm thin sections through the lesion are required. Coronal and sagittal reformatted images are equally obtained to precisely depict the anatomic relationship of the lesion with the adjacent structures. Three-dimensional reformatted images allowing precise pre-surgical planning are usually acquired whenever required by the maxillofacial surgeons or neurosurgeons.

MRI, in addition to having excellent soft tissue contrast, offers the advantages of multiplanar imaging and early detection of marrow oedema, as well as accurate depiction of invasion by tumours and tumour-like lesions [7]. The use of surface coils enhances the usefulness of MRI in evaluating the intracranial connections of small lesions such as dermoid cysts and atretic encephaloceles. Multiphasic contrast-enhanced MRI (CEMRI) enables the assessment of vascular malformations, as well as tumoural and non-tumoural orbital and facial pathology [8]. Diffusion-weighted imaging (DWI) with at least two b values (b = 0 and b = 1000) and calculation of the apparent diffusion coefficient (ADC) facilitates tissue characterisation. Typically, malignant tumours, epidermoid cysts and abscesses show restricted diffusion (high signal on b = 1000 and low signal on ADC). Benign tumours, cysts (except epidermoid) and most vascular malformations show facilitated diffusion (high signal on b = 1000 and high signal on ADC) [7, 9].

F18-Fluoro-deoxy-D-glucose (FDG) positron emission tomography CT (PET-CT) has a limited role, reserved for suspected multifocal disease manifestations or suspected sarcomatous progression of a plexiform neurofibroma into a malignant peripheral nerve sheath tumour (MPNST) [10, 11]. As an alternative to PET-CT, hybrid PET MRI provides robust multiparametric anatomic, functional and metabolic information while significantly reducing radiation exposure [12–16]. It also offers the possibility to accurately detect recurrent disease and to effectively follow patients in a non-invasive fashion by using a combined multiparametric approach once a malignant tumour has been identified [17].

## Primary intraosseous lesions

Common developmental lesions of the facial skeleton include fibrous dysplasia, torus palatinus and torus mandibularis. They present as slowly progressing facial masses and palatal or mandibular palpable indurations. They may occasionally mimic neoplasms on clinical examination.

### Fibrous dysplasia

Fibrous dysplasia (FD) is typically seen in children/adolescents, with a slight predilection for girls. It is a sporadic non-neoplastic disease of the bone-forming mesenchyme in which normal lamellar bone is replaced by immature woven bone with irregular trabeculae [18]. The condition is caused by a defect in the differentiation and maturation of osteoblasts. Histology may show secondary changes, such as aneurysmal-bone cyst-like features, or extensive myxoid changes; there is no nuclear atypia and mitoses are rare [18]. FD leads to progressive enlargement of the affected bone, thus resulting in facial deformity, exophthalmos, visual impairment or paraesthesia due to foraminal compression. In 70–80% of cases, FD is monostotic, and in the remaining 20–30%, it is polyostotic [18, 19]. When FD affects the skull and facial bones alone, the term craniofacial FD is used [19]. Whenever alkaline phosphatase increases dramatically in a patient with polyostotic FD, malignant degeneration should be ruled out.

**Fig. 1:**
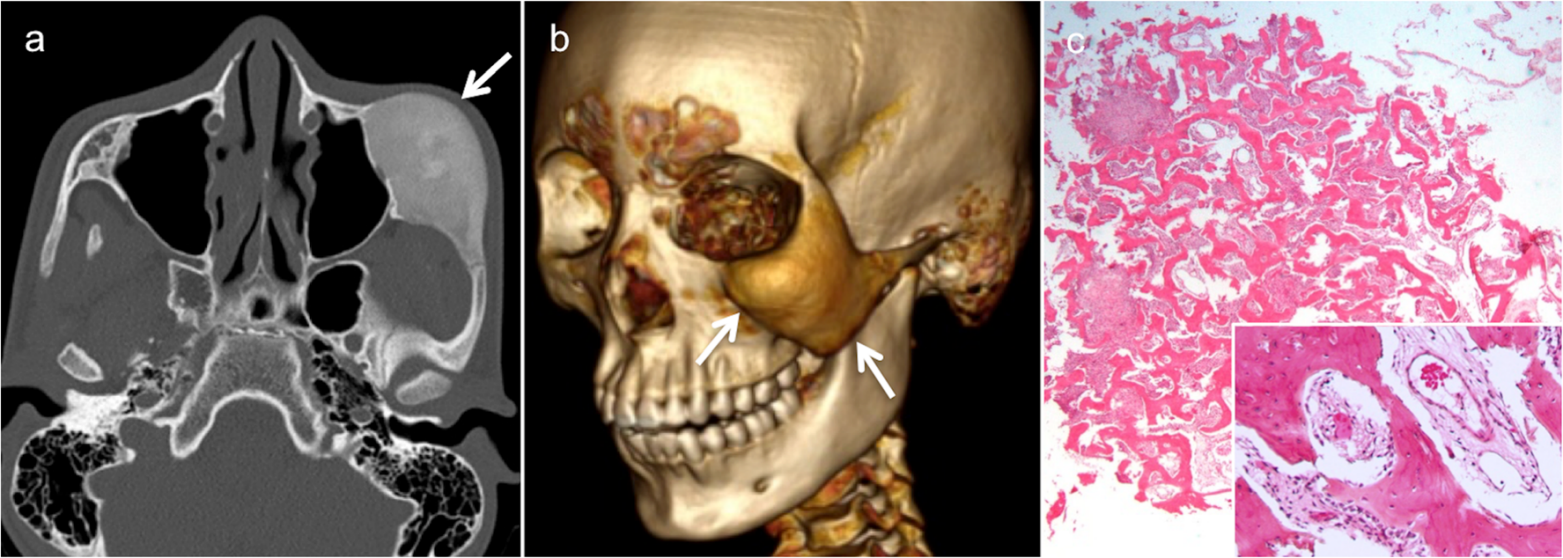
Fibrous dysplasia (FD) of the maxilla in a 12-year-old boy with rapidly increasing facial asymmetry. **a** Axial computed tomography (CT) scan (bone window) shows expansile lesion of the left zygomatic arch with ground-glass opacity (*arrow*). **b** Three-dimensional CT volume rendering (VR) reconstruction depicts zygomatic bone remodelling involving the floor and lateral wall of the orbit and the zygomatic arch (*arrows*). Partial surgery was performed six years later for cosmetic reasons. **c** Low-power photomicrograph obtained after partial lesion resection reveals irregular, curvilinear trabeculae of woven and lamellar bone surrounding fibrous tissue with bland-appearing fibroblastic cells (original magnification, ×100; haematoxylin and eosin [H&E] stain). At higher power, (*inset* in **c**), the bone trabeculae are devoid of osteoblastic rimming (original magnification, ×200)

**Fig. 2:**
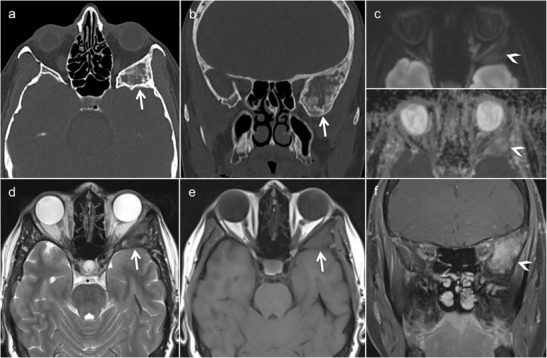
Magnetic resonance imaging (MRI) findings of FD involving the greater wing of sphenoid bone in a patient with left orbital pain. Axial (**a**) and coronal (**b**) CT images (bone window) show an enlarged greater wing of the left sphenoid bone (*arrows*) with medullary expansion and intact cortical outline. Ground-glass opacities within the affected bone. No bone destruction. **c** Diffusion-weighted imaging ((DWI) reveals intermediate signal on the b = 1000 image (*arrowhead*, *upper image part*) and on the ADC map (*arrowhead*, *lower image part*, apparent diffusion coefficient [ADC] = 1.4 × 10^−3^ mm^2^/s). **d** Heterogeneous low signal on T2 without intracranial extension. **e** Homogeneous low T1 signal (*arrow*). **f** Heterogeneous non-specific enhancement on the gadolinium-enhanced coronal fat-saturated T1 (*arrowhead*)

In most patients with FD, CT and CBCT reveal characteristic bony expansion with a “ground-glass” appearance (Figs. 1 and 2) or mixed radiolucency, widened diploic space with outer table displacement or bubbling skull vault lesions (Fig. 2). The fibrous stroma and osteoid material are hypointense on T1, while on T2, the signal intensity may be variable. Marked or heterogeneous enhancement can be seen on CEMRI (Fig. 2). On DWI, FD typically has higher ADC values than malignant bone tumours (ADC mean in FD = 2 × 10^−3^ mm^2^/s); however, lower ADC values are not exceptional and they do not help in the differential diagnosis. On FDG PET-CT, FD may display high metabolism, mimicking malignancy [20]. However, the characteristic ground-glass appearance of FD on CT and, whenever present, high ADC values on diffusion-weighted MRI help in differentiation [18, 19]. Although the aspect of FD on CT/CBCT is most often characteristic, a sclerotic and osteolytic pattern (so-called “pagetoid pattern”) [19] can render differentiation from Paget’s disease difficult. Nevertheless, the latter affects the skull vault of elderly patients and typically spares the facial skeleton. Because of an increased vascularisation of the newly formed bone in Paget’s disease, a “cotton wool” CT appearance and heterogeneous enhancement on MRI are common. Cystic forms of FD may mimic ossifying fibroma, although the latter tends to appear more mass-like, with well-defined borders and more localised than FD [7]. Various intraosseous lesions with heterogeneous contrast enhancement may mimic FD on MRI, such as intraosseous meningioma, giant cell tumour and sclerotic metastases (prostate and lung cancer). The typical age of presentation and the characteristic CT/CBCT aspect, however, make differentiation from these entities straightforward in the paediatric age group. Although radiological findings are very characteristic but not pathognomonic of FD, they must always be correlated with patient demographics and, in rare cases, with histopathology [19].

The management of craniofacial FD includes a “wait and see” policy till the completion of the facial skeletal growth and surgery is performed in cases of persistent or increasing facial deformity (Fig. 1). Because complete surgical excision may lead to aesthetic and functional deficits, a more limited approach to reduce the size of the lesion is often carried out. Nevertheless, in cases with major facial disfigurement or severe optic nerve compression, resection before completion of facial growth is required [21]. Increased risk of malignant degeneration has rendered treatment with radiotherapy obsolete (Table 1).

**Table 1:** Summary of radiologic findings and differential diagnosis

	US	RX	CT/CBCT	MRI	FDG PET	Differential diagnosis
Fibrous dysplasia (FD)	(Not indicated)	Expanded, thickened bone with ground-glass density Occasionally areas of sclerosis or lucency	Expansile bone lesion in the medullary space with variable attenuation: sclerotic FD (ground-glass density), pagetoid FD (mixed radiopacity and radiolucency), cystic FD (centrally lucent lesions with thinned but sclerotic borders)	Low signal on T1 in ossified ± fibrous portions Variable signal on T2. In the active phase, heterogeneous pattern Usually, high ADC values on DWI Fibrous components may enhance intensely	Variable FDG metabolism	Paget disease (adults) Ossifying fibroma Meningioma Metastasis Chondrosarcoma Giant cell tumour
Cherubism	Submandibular lymph node enlargement may be present	Well-defined, bilateral, multilocular, expansile, radiolucent lesions of the jaws in children Symmetrical enlargement of the mandible and maxilla Teeth displacement, absence of dental follicles	Symmetrical, multilocular pseudocysts in the jaws, with few irregular bony septa, usually without other craniofacial involvement Rounded scalloped lesion margins with marked bony expansion	Heterogeneous signal intensity Signal intensity changes in areas that are apparently normal on radiographs or CT	(Not indicated)	Central giant cell granuloma Noonan-like/multiple giant cell lesion syndrome Hyperparathyroidism
Odontogenic keratocyst (OKC) in NBCCS	(Not indicated)	Multiple and expansile cystic masses of the jaws Radiolucent with sclerotic rim Unilocular or multilocular lesions May displace developing teeth, resorb roots of erupted teeth, cause tooth extrusion Rarely resorption of adjacent teeth	Corticated expansile cystic lesions with smooth or scalloped borders Density varies with viscosity of contents No detectable enhancement	Intermediate to high signal on T1 and heterogeneous signal on T2 May have low ADCs due to ortho-/parakeratin and/or haemorrhage No solid enhancing tissue, thin or no enhancing rim	(Not indicated)	Periapical (radicular) cyst Dentigerous (follicular) cyst Ameloblastoma
Torus	(Not indicated)	Areas of increased bony density of variable size arising from the mid-portion of the hard palate (TP) or along the mandibular or maxilla margins (TM and TMax)	Often of fortuitous discovery Typical localisation in oral cavity Bone density of a non-infiltrating exophytic cortical bone lesion, without enhancement	Low signal intensity in T1 and T2 No soft tissue involvement and no enhancement	(Not indicated)	Osteoma
Dermoid and epidermoid cysts	Well demarcated avascular homogeneous mass in epidermoid type Heterogeneous echostructure and minimal increase through transmission ± hyperechoic focus with posterior acoustical shadowing consistent with calcification in dermoid type Pseudosolid appearance	(Not indicated)	*Epidermoid:* low-density, well-demarcated cystic mass with fluid density material inside the lesion *Dermoid:* well-circumscribed cystic mass with fatty, fluid, calcified or mixed contents Typical location, ovoid or tubular morphology Depending on their location, DC may present as osteolytic lesions or may cause scalloping of the near bone	*Epidermoid:* homogeneous fluid signal (high signal on T2), diffuse high signal on T1 if high protein fluid. Typically restricted diffusion on DWI *Dermoid:* heterogeneous high signal on T2 (intermediate signal if fat, focal areas of low signal if calcifications, complex fluid signal on T1). Fatty elements show focal or diffuse high signal on T1 and low signal if fat saturation is used. May have restricted diffusion on DWI. Thin rim enhancement or none	(Not indicated)	*Depending on the location of DC:* *Supraorbital region, floor of the mouth and cheek:* Thyroglossal duct cyst Lymphatic malformation Ranula Abscess *Intraorbital:* Orbital infantile haemangioma Orbital lymphatic malformation Orbital venous malformation *Nose (see below)*
Nasal dermoid sinus cyst (NDSC)	(See above)	No specific x-ray findings on the radiograph of the skull	Midline lesion anywhere from nasal tip to the anterior skull base at the foramen caecum Fluid attenuation cyst and tract from nasal dorsum to skull base within nasal septum Fat-containing mass (dermoid) No specific enhancement Bifid or deformed crista galli or cribriform plate with large foramen caecum Morphology: ovoid mass ± tubular sinus tract	The lipomatous content of the NDSC appears hyperintense on T1 and hypointense on fat-suppressed images (dermoid) Sagittal plane displays course of sinus tract from nasal dorsum to skull base Restricted diffusion on DWI (epidermoid) Rim enhancement or none	(Not indicated)	Nasal glioma Fronto-ethmoidal cephalocele Fatty marrow in crista galli Non-ossified foramen caecum
Plexiform neurofibroma (PNF)	Lobular, serpiginous infiltrative soft tissue mass	Trophic changes and specific signs of bone involvement of the affected structures	Infiltrative trans-spatial appearance Mild contrast enhancement	Target sign: central T2 hypointensity in the multilobulated hyperintense mass Contrast enhancement of infiltrative solid mass (mild to moderate)	Hypermetabolism in sarcomatous transformation	Venous malformation Lymphatic malformation Sarcoma
Venous vascular malformation (VVM)	Variable size of multilobulated, solitary or multiple, spongy and compressible lesion with vascular channels No arterial flow on colour Doppler Hyperechoic foci with posterior acoustic shadowing consistent with phleboliths (if present)	Characteristic phleboliths May show trophic changes of adjacent bones	Circumscribed or trans-spatial, lobulated soft tissue mass, isodense to muscle, with rounded calcifications (phleboliths), often drained by enlarged veins Variable contrast enhancement: patchy and delayed or homogeneous and intense No enlarged feeding arteries Bone remodelling of the adjacent bone	Multilobulated mass with variable signal intensity on T1 Cyst-like appearance of large vascular channels, hyperintense on T2 Smaller vascular channels appear more solid and with intermediate signal intensity Vascular signal voids on T2 due to enlarged dysplastic draining veins Phleboliths appear as rounded or oval signal voids Contrast enhancement is variable, may be mild to intense, delayed, heterogeneous or homogeneous Lymphatic component of VM do not enhance	(Not indicated)	Arteriovenous malformation Lymphatic malformation Infantile haemangioma Dermoid and epidermoid cyst
Lymphatic malformation (LM)	Multicystic paediatric soft tissue mass with fluid–fluid levels Macrocystic LM (> 1 cm): soft, compressible anechoic cystic mass, usually with thin internal septations High echogenicity if haemorrhage or proteinaceous fluid Microcystic LM (< 1 cm): infiltrative solid-appearing mass of subcutaneous soft tissue, mildly hypo- or hyperechoic	(Not indicated)	Multilocular, fluid-attenuation mass, typically uniform, may contain foci of high attenuation due to haemorrhage or protein or low attenuation due to fat or lymph components Subtle fluid–fluid levels of blood products Contrast enhancement of septations in macrocystic LM	Largely bright fluid signal intensity mass with hypointense septa on T2 Fluid–fluid levels due to haemorrhage Bright signal intensity on T1 due to haemorrhage, protein, fat and lymph components Thin rim/septal contrast enhancement of macrocystic lesion. Confluent enhancement of infiltrating tissue in microcystic LM No high-flow vessels intrinsic to lesion	(Not indicated)	Venous malformation Infantile haemangioma Soft tissue sarcoma Soft tissue infection
Cephalocele	Obstetrical US: soft tissue mass through osseous defect	*Fronto-ethmoidal:* midline frontal, intranasal or medial orbital bony defect	Heterogeneous, mixed-density mass variable amounts of CSF and brain parenchyma, extending through bony defect Intrathecal contrast fills subarachnoid space and surrounds soft tissue extending through bony defect (used only when MRI and CT still equivocal) CT cisternography may be useful in localising CSF leak, especially if CSF rhinorrhoea or otorrhoea is present *Fronto-ethmoidal:* crista galli may be bifid or absent. Deficient or absent cribriform plate *Skull base:* depicts osseous defect in skull base *Temporal bone:* focal bone defect in tegmen tympani or mastoid *Petrous apex:* unilateral or bilateral smooth expansile petrous apex lesion due to herniation of posterolateral wall of Meckel cave Enlarged petrous apex porus trigeminal notch	Soft tissue mass isointense to grey matter on T1 Hyperintense signal of CSF surrounds herniated soft tissue parenchyma on T2 Tissue may show hyperintense signal due to gliosis Mass showing contiguity with intracranial brain parenchyma and CSF No abnormal contrast enhancement or mild rim enhancement is noted within soft tissue Meninges may enhance in case of infection or inflammation	(Not indicated)	*Depending on the location of cephalocele:* *Fronto-ethmoidal (frontonasal, nasoethmoidal, naso-orbital type) and skull base (nasopharyngeal, spheno-orbital and sphenomaxillary type):* Nasal glioma Orbital dermoid and epidermoid Nasal dermal sinus Nasolacrimal duct mucocele Teratoma *Temporal bone:* Cholesteatoma with tegmen dehiscence Middle ear cholesterol granuloma Temporal bone arachnoid granulation *Petrous apex:* Petrous apex cholesterol granuloma Petrous apex congenital cholesteatoma Petrous apex mucocele

### Cherubism

Cherubism is a rare autosomal dominant disorder with unknown prevalence. It results from a genetic mutation affecting bone metabolism and remodelling, and at least 15 mutation types have been identified so far [22, 23]. Multilocular pseudocysts progressively replace the lower and upper jaws, usually without other craniofacial involvement. Histology is not specific, showing fibrous tissue and osteoclast-like giant cells [22]. The affected children develop progressive, painless and symmetric enlargement of the mandible and maxilla, and, eventually, the typical cherub face. Dental abnormalities are the most common complications, whereas ophthalmological and respiratory complications are rare [22]. The dentomaxillofacial deformities tend to progress up to adolescence and then regress spontaneously after puberty [24].

**Fig. 3:**
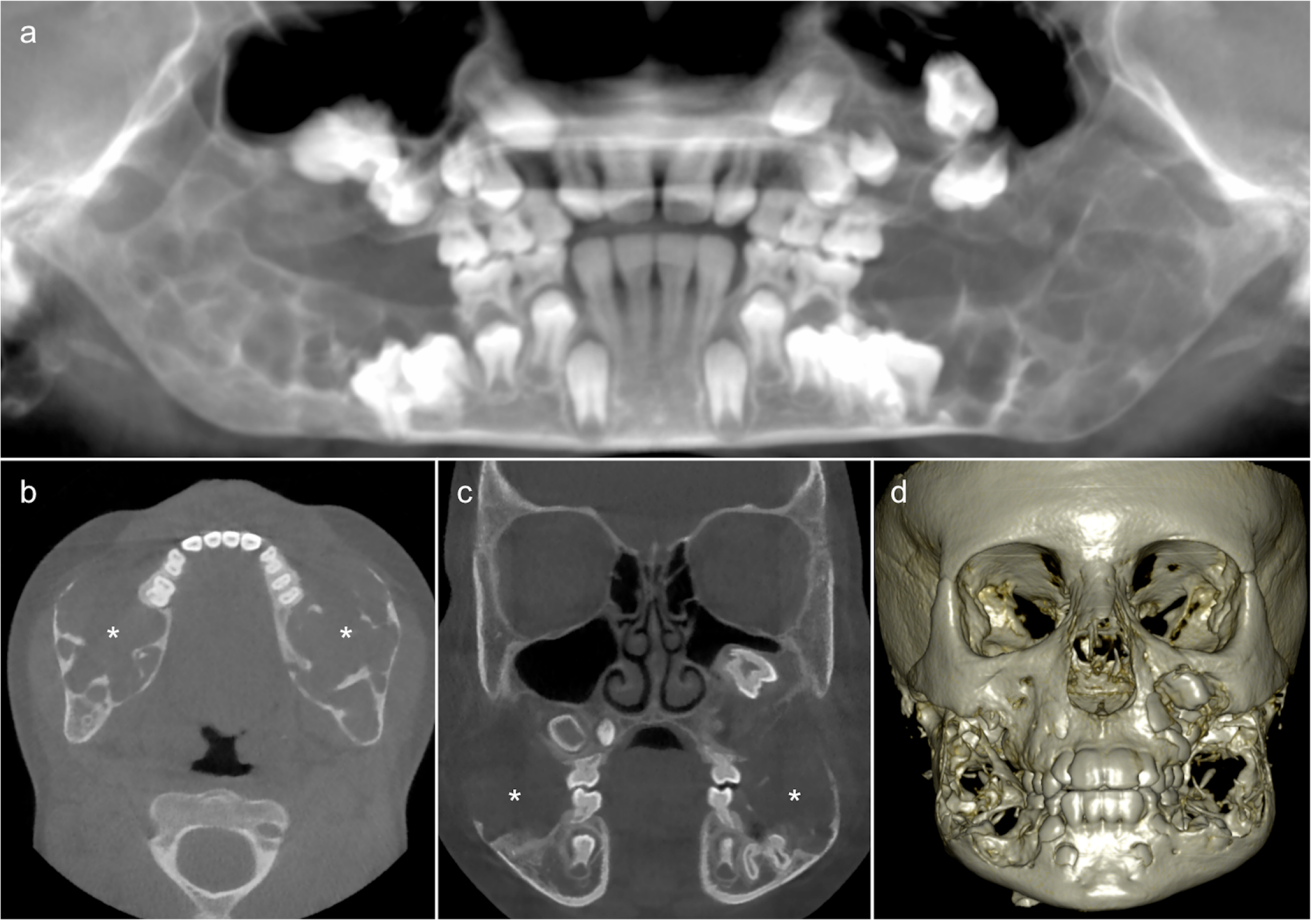
An 8-year-old boy with progressive painless and symmetric bilateral facial enlargement. **a** Translucent cone beam CT (CBCT) thick-slab reconstruction shows well-defined bilateral multilocular radiolucencies with deformation and symmetric bilateral enlargement of the mandible and maxilla, and dental abnormalities (displaced permanent teeth and unerupted first molars). Axial (**b**) and coronal (**c**) CBCT images show multilocular pseudocystic osteolytic lesions with a few irregular bony septa (*asterisks*), no periosteal reaction, teeth displacement and inferior alveolar nerve canal invasion. **d** Hypertrophic osteolytic mandibular and maxillary lesions typical of cherub face as seen on the three-dimensional CBCT reconstruction

OPT shows well-defined bilateral multilocular radiolucent lesions causing symmetrical bilateral enlargement of the mandible and maxilla (Fig. 3). CT/CBCT depicts expansile lytic jaw lesions separated by irregular bony septae, teeth displacement and narrowing of neural foramina (Fig. 3). MRI is reserved for complicated cases to evaluate orbital involvement and airway narrowing [25]. Nevertheless, it has been shown that MRI may reveal additional bone changes not seen on CT or conventional x-ray images [25]. These jaw lesions seen in cherubism have a typical “soap bubble” appearance on CT and OPT; they are of bone-related origin and should not be confused with lesions of odontogenic origin, despite involvement of the teeth and resulting dental abnormalities [5, 26].

The differential diagnosis of cherubism includes Noonan-like/multiple giant cell lesion syndrome, FD and central giant cell granuloma of the mandible and maxilla [27, 28]. Patients with Noonan-like/multiple giant cell lesion syndrome display cherubism-like jaw manifestations, short stature and developmental delay [29]. Central giant cell granuloma is unilocular and cystic, whereas FD causes asymmetric, poorly defined bone expansion, as described above [18]. Although cherubism spontaneously regresses after puberty, orthodontic and ophthalmologic surveillance is recommended.

### Nevoid basal cell carcinoma syndrome

The triad of nevoid basal cell carcinomas, jaw cysts and bifid ribs is known as nevoid basal cell carcinoma syndrome (NBCCS) or Gorlin–Goltz syndrome [30, 31]. NBCCS is a rare autosomal dominant neurocutaneous syndrome caused by mutations in the *PTCH1* gene on chromosome 9p22.3. This multisystemic disorder is characterised by predisposition to multiple basal cell carcinomas (BCCs), odontogenic keratocysts, desmoplastic variant of medulloblastoma (DVM) and skeletal, dental, ophthalmological and neurological abnormalities [32–34]. NBCCS patients have a life expectancy similar to that of the general population, provided that tumours and BCCs are detected and treated early [34].

**Fig. 4:**
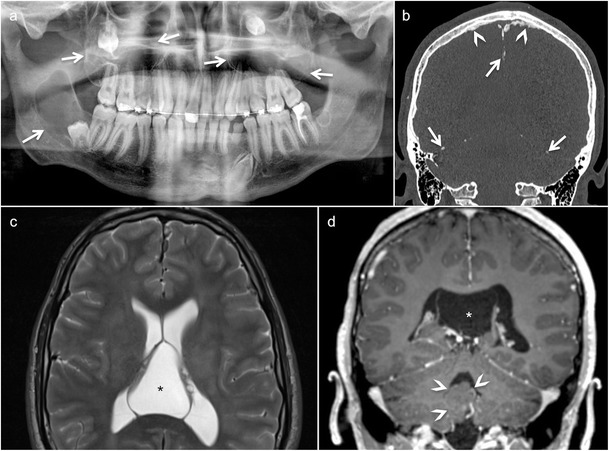
Typical manifestations of nevoid basal cell carcinoma syndrome (NBCCS) in a 16-year-old boy. **a** Orthopantomography (OPT) shows cystic lesions of the mandible and maxilla (*arrows*), with unilocular and multilocular pattern and smooth or scalloped borders associated with displaced and unerupted permanent teeth. **b** Coronal CT scan (bone window) shows ectopic calcifications of the falx cerebri and tentorium cerebelli (*arrows*) and spotted meningeal calcifications (*arrowheads*). Brain MRI reveals a cavum veli interpositi on axial T2 (*asterisk* in **c**) and coronal contrast-enhanced T1 (*asterisk* in **d**) and also vermian dysgenesis (*arrowheads* in **d**)

**Fig. 5:**
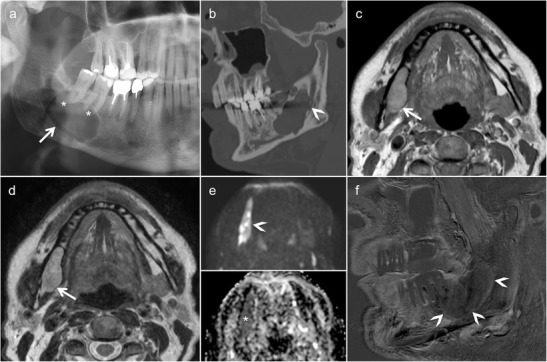
Characteristic radiological findings of odontogenic keratocyst (OKC) as seen in an elderly male. **a** OPT shows a large, well-defined osteolytic lesion involving the body and angle of the mandible on the right encompassing the right inferior alveolar nerve canal (*arrow*). Absence of root resorption (*asterisks*). **b** Multiplanar reformatted CT image (bone window) shows an unilocular cystic expansile lesion extending into the right inferior alveolar nerve canal (*arrowhead*). No periosteal reaction or pathological fracture. T1 (**c**) and T2 (**d**) axial images reveal thinned cortex (*arrows*) with postero-inferior cortical breach. **e** DWI reveals high signal on the b = 1000 image (*arrowhead*, *upper image part*) and low signal on the ADC map (*asterisk*, *lower image part*, ADC = 0.7 × 10^−3^ mm^2^/s), compatible with restricted diffusion due to intralesional ortho-/parakeratin accumulation and/or haemorrhage. **f** Subtraction image (T1 post-contrast and T1 pre-contrast) in the sagittal oblique plane shows only a thin enhancing lesion rim (*arrowheads*)

Odontogenic keratocysts (OKCs), formerly called keratocystic odontogenic tumours [26], are thought to originate from the dental lamina; OKCs are the hallmark of NBCCS [5, 32]. They are seen in 75% of NBCCS patients. Multiple OKCs occur in most patients before the age of 10 years, with a peak incidence in the second and third decades of life. Clinically, the lesions are asymptomatic until they become large enough to cause jaw swelling. Common locations include the mandibular molar-ramus region (44% of cases) and the mandibular incisor-canine region (18%) [32]. Malignant degeneration into ameloblastoma or squamous cell carcinoma has been reported [35–37]. OPT and CT/CBCT in OKCs typically show unilocular or multilocular cystic lesions with smooth or scalloped borders (Figs. 4 and 5). Although OKCs are the most consistent and representative lesions of NBCCS in childhood [33], they can incorporate the crown of an unerupted and/or displaced tooth, mimicking dentigerous cysts [5]. Due to their extension into the soft tissues and the possibility of tooth resorption, the differentiation of OKC from ameloblastoma can be very challenging, if not impossible, on OPT, CT or CBCT. On MRI, the high signal on T2 and the weak enhancement of the thin and regular walls in OKC is very useful to differentiate these entities from multicystic forms of ameloblastoma; the latter typically show solid nodular components and irregular thick septae [5].

Treatment options in OKCs include surgical enucleation and cryotherapy. The reported recurrence rate after enucleation is as high as 60% [34]. NBCCS patients tend to have associated craniofacial skeletal abnormalities, such as high arched palate, prominent palatine ridges, cleft lip/palate, prognathism and mandibular coronoid process hyperplasia [32, 34]. Further anomalies include parietal/temporal bossing (50%), macrocephaly (40%) and brachycephaly.

CT and MRI of the central nervous system (CNS) in patients with NBCCS typically reveal ectopic calcifications of the falx cerebri and tentorium cerebelli, bony bridging of the sella turcica, spotted meningeal calcification, corpus callosum agenesis/dysgenesis with/without lipoma and vermian dysgenesis (Fig. 4). Other coexisting conditions include congenital communicating hydrocephalus, DVM or other brain tumours (meningioma, oligodendroglioma, glioblastoma, craniopharyngioma) [33, 34]. As opposed to classical medulloblastoma, DVM, which occurs in the first 2 years of life, has a more favourable prognosis [33, 34]. The tumour usually presents with hydrocephalus and tends to grow directly into the brainstem. It is hypointense on T1, hyperintense on T2 and may have characteristic peripheral cysts. Enhancement can be homogeneous/heterogeneous, multinodular or star-shaped and radiating. Because of the hypercellular nature, DVMs show low ADCs (0.629 ± 0.058 × 10^−3^ mm^2^/s), therefore, allowing differentiation from glial tumours, which tend to have high ADCs [38].

Conventional x-rays may detect other sites of musculoskeletal involvement, such as polyostotic bone cysts and flame-like finger lesions (hamartomas), bifid, fused, splayed or missing ribs, scoliosis, spina bifida occulta, pectus deformities, polydactyly, syndactyly and Sprengel type scapula involvement [33]. As suggested recently, radiological work-up in suspected NBCCS comprises brain MRI, OPT, cardiac and abdominal US, and skeletal survey. Major diagnostic criteria for NBCCS include multiple BCCs or one BCC before 20 years of age, jaw OKCs before 20 years of age, palmar/plantar pits, falx cerebri calcification, medulloblastoma and first-degree relatives with NBCCS. Minor criteria include rib anomalies, cleft lip/palate, other skeletal malformations, macrocephaly and ovarian/cardiac fibroma. One major criterion and molecular confirmation, two major criteria or one major and two minor criteria favour the diagnosis of NBCCS [34].

### Torus palatinus, torus mandibularis and torus maxillaris

**Fig. 6:**
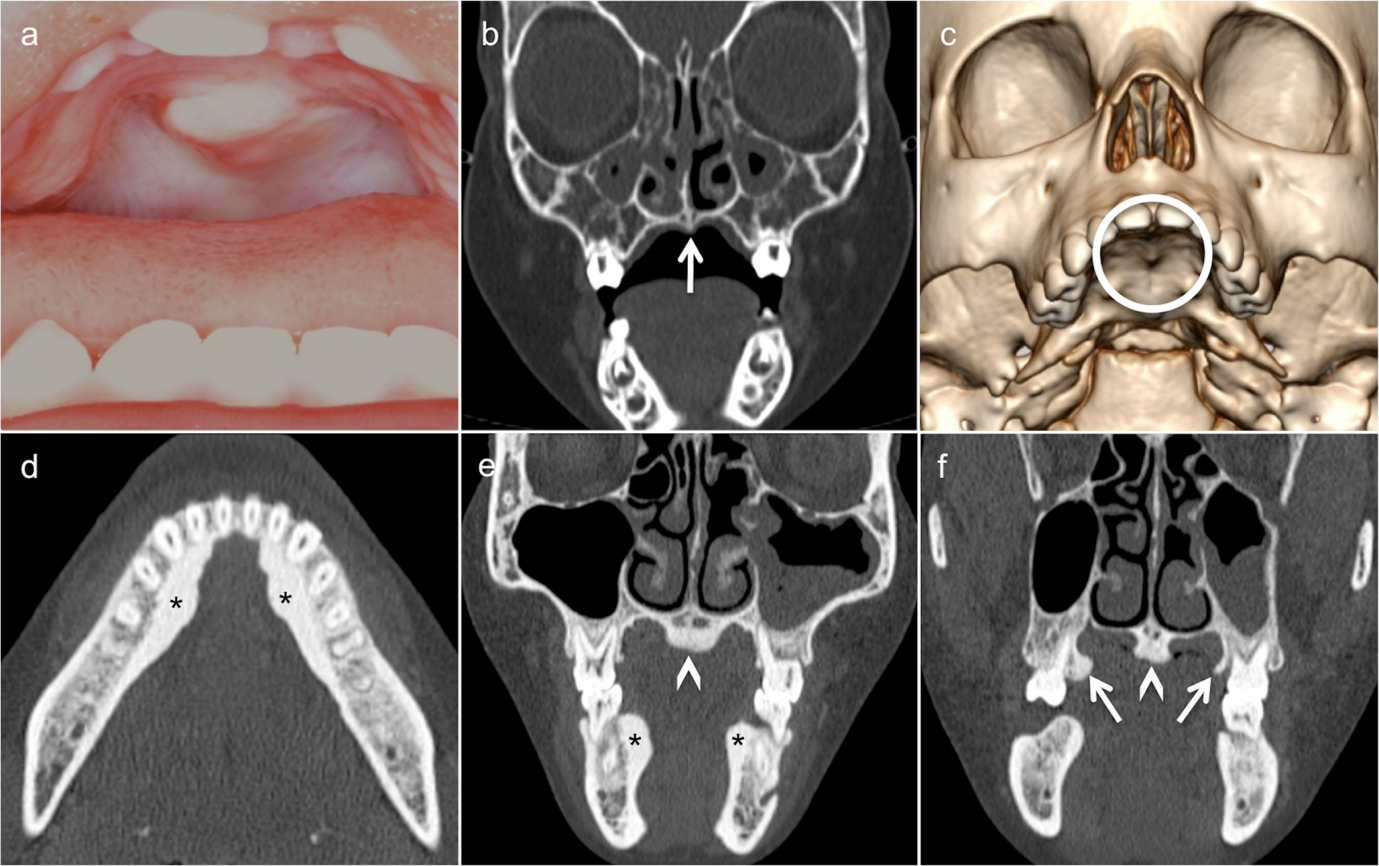
Torus palatinus (TP) in a 3-year-old girl who underwent imaging because of an indurated palpable midline mass of the hard palate increasing in size (**a**) after a fall occurring from a swing hanging on a tree two weeks earlier. Coronal low-dose CT image (bone window) (**b**) and three-dimensional CT VR reconstruction show a small midline spur/exostosis (*arrow* and *circle*). Characteristic aspect of a torus mandibularis (TM) (*asterisks* in **d** and **e**), of a TP (*arrowheads* in **e** and **f**) and of a torus maxillaris (TMax) (*arrows* in **f**) as seen in a young adult. It is worthwhile mentioning that TM and TMax are extremely rarely diagnosed in children

Torus palatinus (TP) is an exostosis occurring along either side of the midline suture of the hard palate. Torus mandibularis (TM) is an exostosis occurring along the lingual surface of the mandible, whereas torus maxillaris (TMax) occurs on the palatal or vestibular side of the alveolar process of the maxilla. According to anthropological studies, tori appear to be more common among populations living in the northern hemisphere as compared to those residing in the south [39]. Although both tori clinically present around the age of 20 years, they are thought to be detectable during infancy too. Since both tori present as an intraoral swelling covered by intact mucosa, they may constitute a diagnostic dilemma (Fig. 6). Although tori usually have no clinical significance, they can be a cause for major parental concern. Most tori do not undergo surgery unless they interfere with dentition and function. The radiologic aspect is straightforward on CT/CBCT or MRI, which depicts a classical exostosis in a typical location (Fig. 6). Although TM and TMax are often bilateral, they may be unilateral or asymmetric, thereby rendering the diagnosis more challenging [40]. Based on radiological findings, it is difficult to misinterpret tori as malignant bone lesions considering their typical aspect and position in association with absent involvement of the overlying soft tissues. However, in some cases, the soft tissues covering the bone growth can become ulcerated and the torus is perceived as new and worrisome, either by the patient him-/herself or at clinical examination.

### Dermoid cysts

**Fig. 7:**
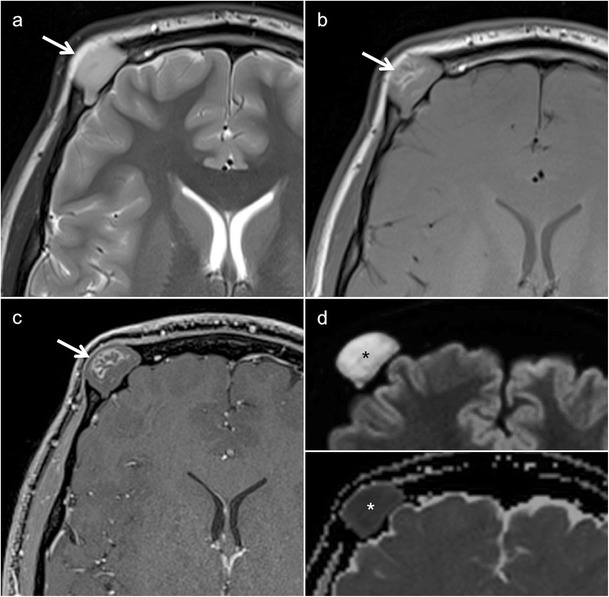
Intraosseous dermoid cyst (DC) in a 17-year-old male presenting as a gradually enlarging right supraorbital swelling since three years. **a** Cystic lesion in the right frontal bone (*arrow*) with non-lipomatous contents on axial T2. On T1 (**b**) and fat-saturated contrast-enhanced T1 (**c**), the lesion appears hypointense and displays intracystic serpiginous hyperintense areas which correspond to haemorrhage, high protein content or saponification (*arrows*). **d** DWI reveals high signal on the b = 1000 image (*asterisk*, *upper image part*) and low signal on the ADC map (*asterisk*, *lower image part*, ADC = 0.6 × 10^−3^ mm^2^/s), compatible with restricted diffusion and characteristic of an epidermoid cyst

Although rare, together with Langerhans cell histiocytosis (LCH), dermoid cysts (DCs) are the most common lesions of the paediatric skull. They result from epithelial sequestration during midline union of the embryonic first and second branchial arches. The generic term “dermoid cysts” comprises three entities: (1) epidermoid cyst (stratified keratinised epithelial lining without skin appendages), (2) true dermoid cyst (stratified keratinised epithelial lining with skin appendages) and (3) teratoma (showing tissues derived from the primitive germ layers) [41]. Only about 7% of DCs are found in the head and neck region, mainly in the supraorbital region, followed by the floor of the mouth, nose, orbit and cheek [41–43]. Common locations of intraosseous DCs include the frontal bone (Fig. 7), maxilla and mandible [42, 44, 45].

**Fig. 8:**
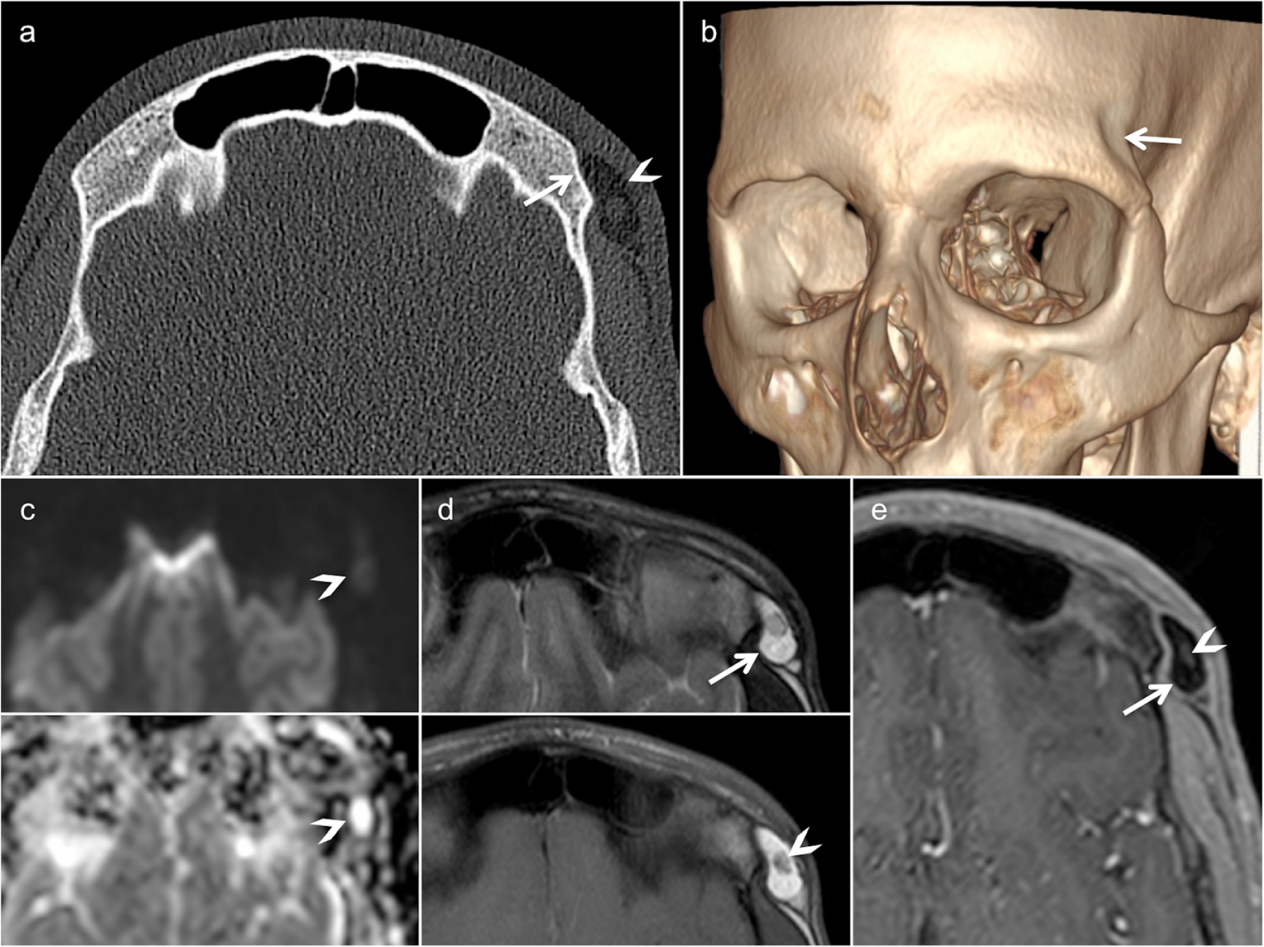
DC with bone remodelling in a 14-year-old male with lateral supraorbital soft tissue swelling and induration. **a** Axial CT (bone window) shows well-demarcated cystic subcutaneous lesion (*arrowhead*) with frontal bone scalloping and no cortical erosion (*arrow*). **b** Three-dimensional CT VR reconstruction shows bone remodelling in the supraorbital left area (*arrow*). **c** DWI reveals high signal on the b = 1000 image (*arrowhead*, *upper image part*) and high signal on the ADC map (*arrowhead*, *lower image part*, ADC = 2.5 × 10^−3^ mm^2^/s), compatible with no restricted diffusion and characteristic of a DC. **d** Peripheral lipid density on T2 (*arrow*, *upper image part*) and T1 with low signal (*arrow* in **e**) in gadolinium-enhanced coronal fat-saturated T1. Central soft tissue density on T1 (*arrowhead* in *lower image part*) with no contrast enhancement (*arrowhead* in **e**), corresponding to squamous debris

DCs are slow-growing lesions causing bone scalloping when arising in immediate bone vicinity (Fig. 8). When arising within the bone, they present as well-delineated osteolytic lesions with sclerotic borders expanding the outer table. On CT, intraosseous DCs may show fat or fluid attenuation and internal calcification, whereas epidermoid cysts have fluid density with no fat contents or calcifications [46]. In true dermoids, fatty components are often collected in nodules, thus giving a “sack-of-marbles” appearance [43]. On MRI, the identification of fat within the lesion differentiates DC from LCH or other intraosseous lesions [7]. Diffusion in DCs may be variable, and the epidermoid subgroup typically shows restricted diffusion (Fig. 7). Small asymptomatic intraosseous DCs do not require immediate treatment, whereas larger lesions may require surgery. When surgery is carried out, complete excision without rupturing the DC is mandatory to avoid recurrence or inflammation.

### Nasal dermoid sinus cyst

Nasal dermoid (or dermal) sinus cyst (NDSC) includes all nasal lesions containing stratified squamous epithelium (ectoderm derivatives) and adnexal structures (mesoderm derivatives) [47]. It is the most common midline congenital nose lesion in children, followed by glioma and encephalocele [48]. NDSC is usually visible at birth or in early childhood. Familial cases and association with other congenital craniofacial anomalies have been described [48–50]. The non-compressible swelling is localised anywhere from the glabella to the columella, and is associated with a sinus opening with intermittent secretions of sebaceous material. Hair protruding through a punctum over the nasal dorsum is pathognomonic; however, this feature is present in less than 50% of patients [47]. The cysts and sinuses may connect with an intracranial component via an abnormal foramen caecum. NDSC are, therefore, classified as superficial, intraosseous, intracranial extradural and intracranial intradural [49, 50]. Complications include recurrent infections, meningitis and brain abscess [48].

**Fig. 9:**
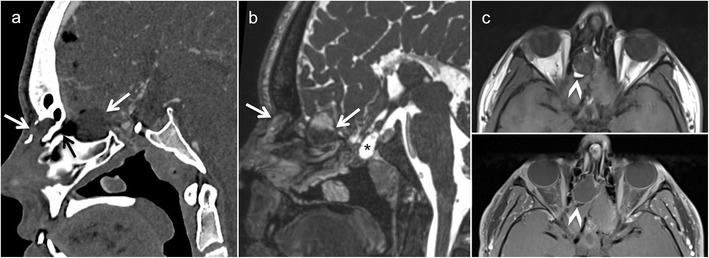
Nasal dermoid sinus cyst (NDSC) visible since birth in a 15-year-old boy. **a** Sagittal contrast-enhanced CT scan (soft tissue windows) shows the extra- and intracranial dermoid cysts (*white arrows*), which connect with each other via the abnormal foramen caecum (*black arrow*). **b** High-resolution sagittal T2 shows that the cystic lesions have different signal intensities (*arrows*), suggesting different proteinaceous/lipomatous contents. Note also corpus callosum agenesis and posterior ethmoidal meningocele (*asterisk*). **c** The lipomatous content (*arrowheads*) of the NDSC appears hyperintense on T1 (*upper image part*) and hypointense on fat-saturated contrast-enhanced T1 (*lower image part*), respectively

High-resolution CT and thin-section MRI (Fig. 9) are essential for preoperative surgical planning, and they must be performed to exclude involvement of the paranasal sinuses and intracranial extension [49]. Indirect signs of intracranial extension include bifid or deformed crista galli, widened foramen caecum and cribriform plate defect [49]. Contrast-enhanced MRI (Fig. 9) is essential for the differentiation between non-enhancing dermoid cyst, enhancing nasal mucosa and other masses, such as haemangioma, meningioma or teratoma [47]. Potential diagnostic pitfalls, especially on MRI, include normal fat deposition occurring during normal bone maturation and during frontal sinus pneumatisation. These fatty changes should not be mistaken for NDSC [51]. A further diagnostic pitfall consists in misinterpreting the normal crista galli as an NDSC. Due to the vicinity of the crista galli to the foramen caecum, it is easy to confuse the high signal intensity of the crista galli on MRI with a dermoid cyst with intracranial extension [51]. The treatment of NDSC requires complete surgical excision, including any associated sinus tract [48].

## Masses with secondary bone involvement

Several developmental lesions of the face or genetic conditions with subsequent development of tumours may lead to bone remodelling and scalloping of the craniofacial skeleton, thereby leading to severe functional problems. These lesions include DCs (see above), vascular malformations (VMs) and plexiform neurofibroma (PNF) [52].

### Plexiform neurofibroma

Neurofibromas are benign peripheral nerve sheath tumours with the nerve of origin usually incorporated within the lesion [53]. Neurofibromas include localised neurofibroma, diffuse neurofibroma and PNF [54]. Localised neurofibromas are solitary lesions which have no association with neurofibromatosis type 1 (NF1, von Recklinghausen’s disease) [55]. PNF is the hallmark of NF1 and is found in 30% of NF1 patients. PNF arises from major nerve branches and is characterised by diffuse long segment nerve involvement [55]. Clinically, PNFs present as subcutaneous masses with a “bag-of-worms” consistency on palpation. PNFs have the potential for malignant transformation in up to 10% of NF1 cases [56]. Whenever PNF shows an abrupt increase in size, a malignant transformation to MPNST needs to be ruled out [57].

**Fig. 10:**
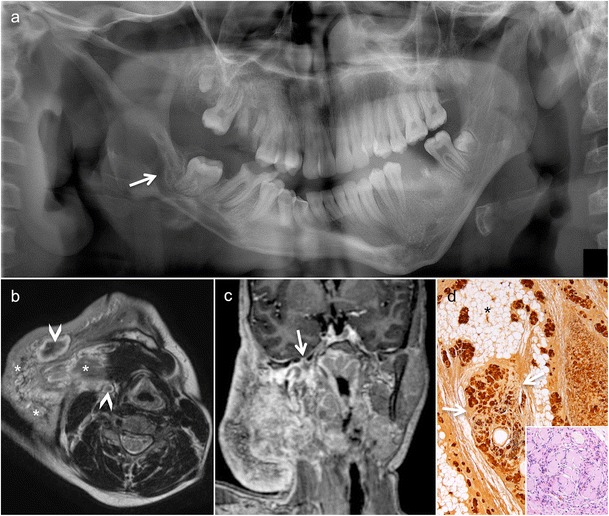
Rapidly enlarging painful plexiform neurofibroma (PNF) in a 15-year-old girl with neurofibromatosis type 1 (NF1). Imaging was performed before PNF resection. **a** Panoramic view shows mandible deformity (thinning and bowing of the right ascending ramus of the mandible and the right mandibular body) with involvement and enlargement of the right inferior alveolar nerve canal (*arrow*). **b** The axial T2 images reveal extensive PNF (*asterisks*) with a characteristic “target sign”: central T2 hypointensity within the multilobulated hyperintense mass (*arrowheads*). **c** Gadolinium-enhanced coronal fat-saturated T1 shows large, infiltrative and multilobulated PNF involving the entire hemiface with perineural spread and extension to the right foramen ovale (*arrow*). **d** Immunohistochemistry (original magnification, ×40; S100 protein) obtained from surgical specimen highlights plexiform nerve bundles (*arrows*) dissecting the adipose tissue of the hypodermis (*asterisk*). Numerous confluent Wagner-Meissner bodies are seen (*inset*, original magnification, ×200; H&E stain)

PNF of the orbit, face and the temporal region is a rare but devastating complication of NF1. The disease is unilateral and leads to progressive bowing and scalloping of the maxillofacial skeleton (Fig. 10). Buphthalmos and sphenoid wing dysplasia (genetically determined) further contribute to vision loss, as there is prolonged compression and stretching of the optic nerve. PNFs of the orbit, temporal region and face are seen as large conglomerate masses on cross-sectional imaging. Sphenoid wing dysplasia, lambdoid suture defects, deformation and scalloping of the mandible are typical imaging findings, making the diagnosis of PNF in NF1 straightforward (Fig. 10) [58]. PNF shows mixed or increased diffusion on ADC maps and diffusion tensor imaging with tractography reconstruction can accurately detect displacement, stretching or interruption of nerve fascicles. FDG PET-CT is a sensitive and specific tool to detect sarcomatous transformation [59]. Sarcomatous transformation should be equally suspected in PNF with rapid increase in size.

As PNF can mimic a venous malformation on MRI, it is important to recognise the classic “target sign” of PNF (central T2 hypointensity within a multilobulated hyperintense mass). This characteristic aspect should not to be confused with phleboliths. Occasionally, PNF may be misdiagnosed as lymphatic malformation (LM) if no iv contrast material is administered. Unlike LM, PNF shows mild to moderate enhancement of the infiltrative and solid mass on contrast-enhanced CT and CEMRI.

### Vascular malformations

#### Venous vascular malformations

Vascular malformations (VMs) of the face are rare congenital anomalies that do not regress over time and which may rapidly enlarge following trauma or endocrine changes. They are subdivided into capillary, venous, arterial and lymphatic malformations. Arterial vascular malformations (AVMs) are high-flow malformations with tortuous arteries and enlarged veins, resulting in serpiginous flow voids on T1 and T2. Capillary and venous vascular malformations (VVMs) are low-flow lesions with patchy to intense contrast enhancement and, occasionally, phleboliths. If the tissues under the skin are affected, the superficial, subcutaneous lesions may appear as slightly blue-coloured skin stains. However, most often, VVMs present as non-pulsatile compressible soft tissue swellings. As VVMs are present at birth, they usually grow with the child and may have long-term cosmetically and functionally disabling consequences, including pain, respiratory compromise and disfigurement. If the lesions are deeply located, there is only slight facial asymmetry, but no colour change. During a Valsalva manoeuvre, VVMs typically enlarge. The slow venous flow predisposes to repeated episodes of intralesional thrombosis, and sudden enlargement may occur [60]. Increased D-dimers have been reported in 42% of patients with VVMs and are highly correlated with pain caused by thrombosis [60]. Depending on patient symptoms, treatment includes multiple procedures (surgery, laser therapy and sclerotherapy).

**Fig. 11:**
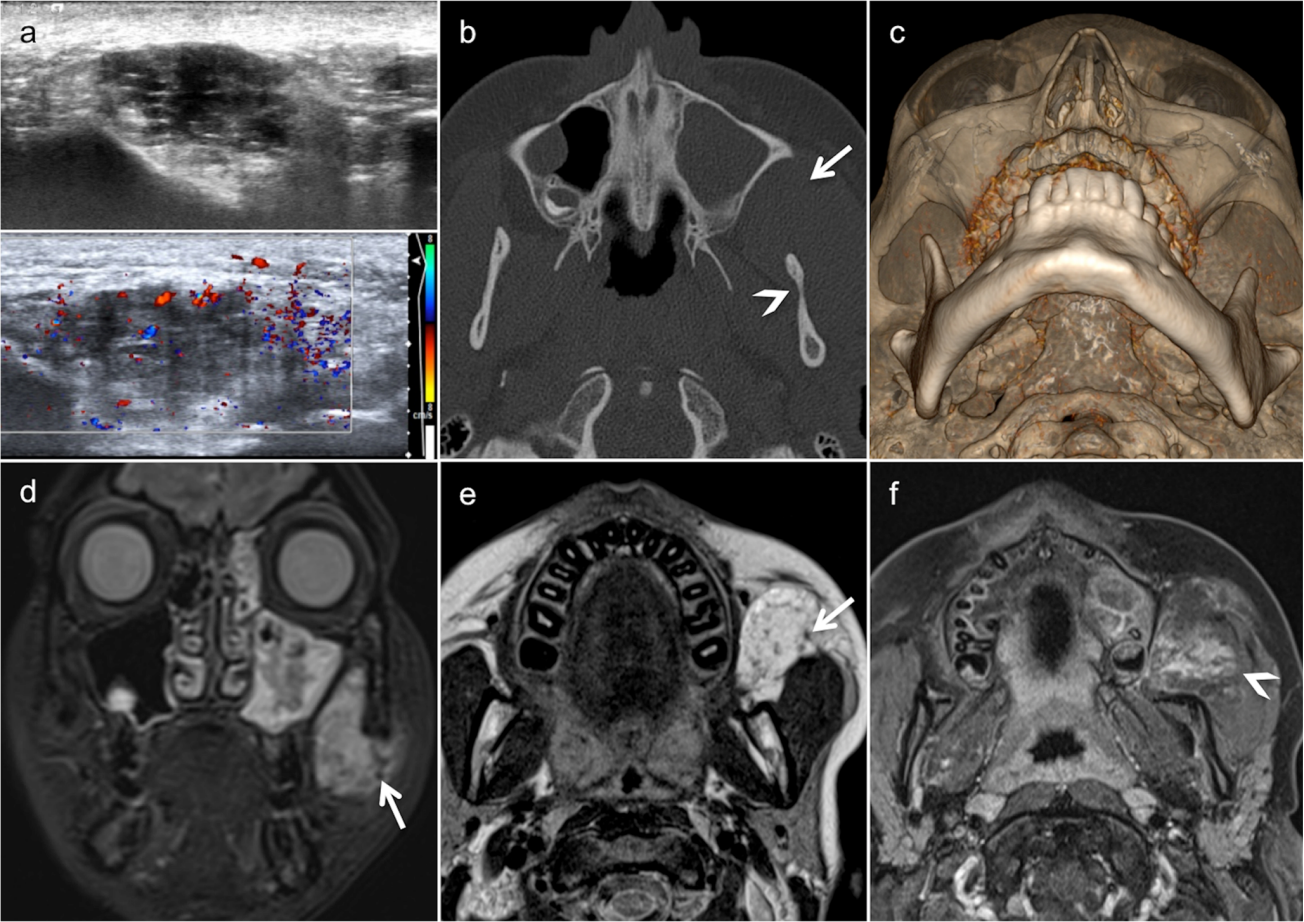
Venous vascular malformation (VVM) in a 12-year-old girl complaining of increasing left cheek swelling causing major facial asymmetry and dental malocclusion. **a** Grey-scale ultrasound (*upper image part*) and corresponding Doppler US (*lower image part*) show hypoechoic and infiltrative mass with poor vascularisation. **b** Axial low-dose CT (bone window) shows shortened and remodelled left ascending ramus of the mandible (*arrowhead*) and increased distance (*arrow*) between the left ascending ramus of the mandible and posterior wall of the left maxillary sinus, compatible with a slow-growing lesion. **c** This increased distance is also clearly seen on the three-dimensional CT VR reconstruction. STIR (**d**) and T2 (**e**) images show a hyperintense mass with multiple small phleboliths (small, dark, rounded areas) situated within Bichat’s fat (*arrows*). **f** Contrast-enhanced axial fat-saturated T1 shows partial, progressive, centripetal lesion enhancement (*arrowhead*) characteristic of a VVM

The diagnosis of VVMs is made with colour Doppler US and with MRI (Fig. 11). On US, VVMs are often compressible and hypoechoic, and they present a heterogeneous sponge-like echotexture. Calcified phleboliths, although pathognomonic, are present only in 16% of cases [60]. Monophasic flow on colour Doppler US is seen in 78% of the cases, whereas absent flow due to thrombosis or sluggish flow below the limits of detection can be seen in 16% of lesions and represents a source of diagnostic confusion [60]. Arterial flow within the VVM is identified in 6% and is either caused by facial arteries traversing the VVM or by Masson tumour [60]. Masson tumour (intravascular papillary endothelial hyperplasia) is an unusual benign lesion thought to represent an atypical form of thrombus organisation [61]. On MRI, VVMs are hypointense on T1 and they occasionally display hyperintense areas caused by intralesional haemorrhage or fatty deposits. The lesions are strongly hyperintense on T2 and show variable patterns of enhancement: homogeneous/heterogeneous, slight/strong, rapid/delayed (Fig. 11). Phleboliths are present as round areas of signal voids on T1-, T2- and contrast-enhanced T1, and they can be confirmed on T2* due to the presence of calcifications and haemosiderin (Fig. 11) [60]. The absence of phleboliths, however, does not exclude a VVM. Following trauma or spontaneous haemorrhage, VVMs may lose their typical imaging appearance due to haematoma or thrombosis [60]. On CT, remodelling of the facial bones is common in larger lesions. Although phleboliths may be visible on conventional x-ray examinations, US and MRI, they are much better recognised on CT. Nevertheless, as mentioned above, phleboliths can be absent in VVMs, whereas rounded calcifications can also be seen in DCs. These calcifications should not be confused with phleboliths. Further diagnostic pitfalls include combined venous and lymphatic malformations and/or VVMs with large vascular channels that appear cyst-like on MRI. On MRI, the presence of vascular signal voids, which are atypical for VVMs, is due to enlarged dysplastic draining veins; the latter should not be confused with high-flow vascular lesions, i.e. AVM.

#### Lymphatic malformations

Lymphatic malformations (LMs; formerly called lymphangiomas or cystic hygromas) are benign vascular lesions, which tend to vary in size, location and extension [62, 63]. They are thought to be a developmental anomaly of the lymphatic system in which the drainage to the venous system is either poorly developed or absent, leading to stagnation of lymph with subsequent expansion and proliferation of the lymphatic system [64]. LMs can be subdivided into three types: macrocystic (cyst diameter > 0.5 cm), microcystic (smaller lymphatic channels permeating subcutaneous tissues) and combined (macro- and microcystic) [62]. The reported incidence of 1.2–2.8% is likely to be underestimated, as deep-seated LMs often remain undetected. Approximately 50–75% of LMs are present at birth, of which 80–90% are detected before 2 years of age and few on antenatal US. LMs, which are occult and asymptomatic at birth, may enlarge with hormonal changes, and secondary to trauma or infection [63]. Of all LMs, 45–52% occur in the head and neck region (oral cavity, orbit and deep neck spaces).

Clinical presentations of LMs are related to infection, haemorrhage and mass effect. Mass effect can cause displacement of vital structures, thus impairing vision, breathing or swallowing. Juxtaposed bones show scalloping, sutural widening and remodelling. Trauma and infection may cause spontaneous bleeding or purulent discharge.

**Fig. 12:**
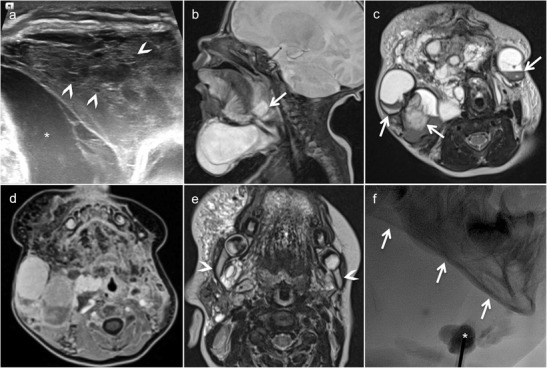
Lymphatic malformation (LM) in a term-born-boy presenting with a large anterior and lateral neck mass beneath a bluish-coloured skin. **a** Ultrasound of the neck shows that the mass is composed of large fluid-filled macrocystic (*asterisk*) and microcystic (*arrowheads*) portions. **b** Sagittal T2 illustrates the macrocystic submental component extending into the base of the tongue (*arrow*). Percutaneous sclerotherapy was carried out. Post-sclerotherapy follow-up MRI (**c**, **d**). **c** Axial T2 depicts intracystic fluid/fluid levels due to internal bleeding (*arrows*). Fat-saturated, contrast-enhanced T1 (**d**) shows enhancement of the thin septae of the macrocystic and microcystic components. Two years later, the mandibular deformation is visible on the follow-up MRI (T2W image, **e**, *arrowheads*) and on an x-ray image (**f**, *arrows*), obtained during percutaneous sclerotherapy (*asterisk*)

Optimal evaluation of superficially located LMs is possible with Doppler US. Uncomplicated LMs appear as anechoic cystic lesions with internal septations and no vascularity on colour Doppler US. US allows differentiation between LM-LMVs and venous malformations, as well as other mixed vascular lesions [64]. CT and MRI accurately delineate the location, extent and size of LMs. On CT, LMs appear as low attenuation cystic lesions. On MRI (Fig. 12), cystic LMs display intermediate or low signal intensity on T1 and high signal intensity on T2. Intralesional fluid–fluid levels due to spontaneous haemorrhage show intermediate or high signal intensity on T1 and low signal intensity on T2. In the case of infection, DWI demonstrates restricted diffusion. LMs display slight contrast-material enhancement along cyst walls and internal septae, without opacification of dilated lymphatic channels, as opposed to slow-flowing vascular malformations, which show centripetal progressive enhancement and phleboliths (Fig. 12). As LMs may show spontaneous regression in very young children, whenever possible, treatment should be delayed until the age of 2–3 years [64]. The management of complex cases can be challenging and requires a multidisciplinary approach. It often involves a combination of surgical resection, sclerotherapy and laser therapy [62].

### Cephalocele

A cephalocele is a generic term applied to several congenital or acquired conditions. Encephalocele is herniation of brain parenchyma through an osseous-dural defect of the skull base or cranial vault, whereas meningocele is herniation of meninges alone. Meningoencephalocele comprises herniation of brain parenchyma along with meninges, whereas a meningoencephalocystocele includes herniation of ventricle besides the other two [65]. Gliocele implies herniation of gliotic brain parenchyma. Concomitant CSF leak with these conditions is common [66]. The estimated incidence of cephalocele is 0.8–4/10,000 live births, with a well-recognised geographical variation between subtypes and female preponderance [67, 68]. Congenital cephalocele is caused by closure defect of the neural tube or by a focal failure of cartilage formation or ossification of the skull base. Common locations are the occipital and parietal bone, frontoethmoidal junction, and sphenoid and temporal bone [69]. Although congenital cephaloceles are present at birth, they may remain clinically occult until adulthood. The clinical presentation depends on the amount of herniated cerebral tissue, on the topography of the lesion and on the associated cerebral and craniofacial anomalies [70]. It includes CSF rhinorrhoea, facial deformity, pulsating proptosis and exophthalmos, middle ear effusion, conductive hearing loss, headache, epilepsy and ascending infections (osteomyelitis, meningitis, encephalitis and abscesses). Migrational abnormalities, corpus callosum agenesis, cleft lip and palate, coloboma and microphthalmia are common concomitant developmental anomalies [68]. Naso-ethmoidal encephalocele should be differentiated from nasal glioma, which is a congenital non-neoplastic lesion composed of dysplastic glial tissue usually not connected to the brain and without direct communication with the subarachnoid spaces.

**Fig. 13:**
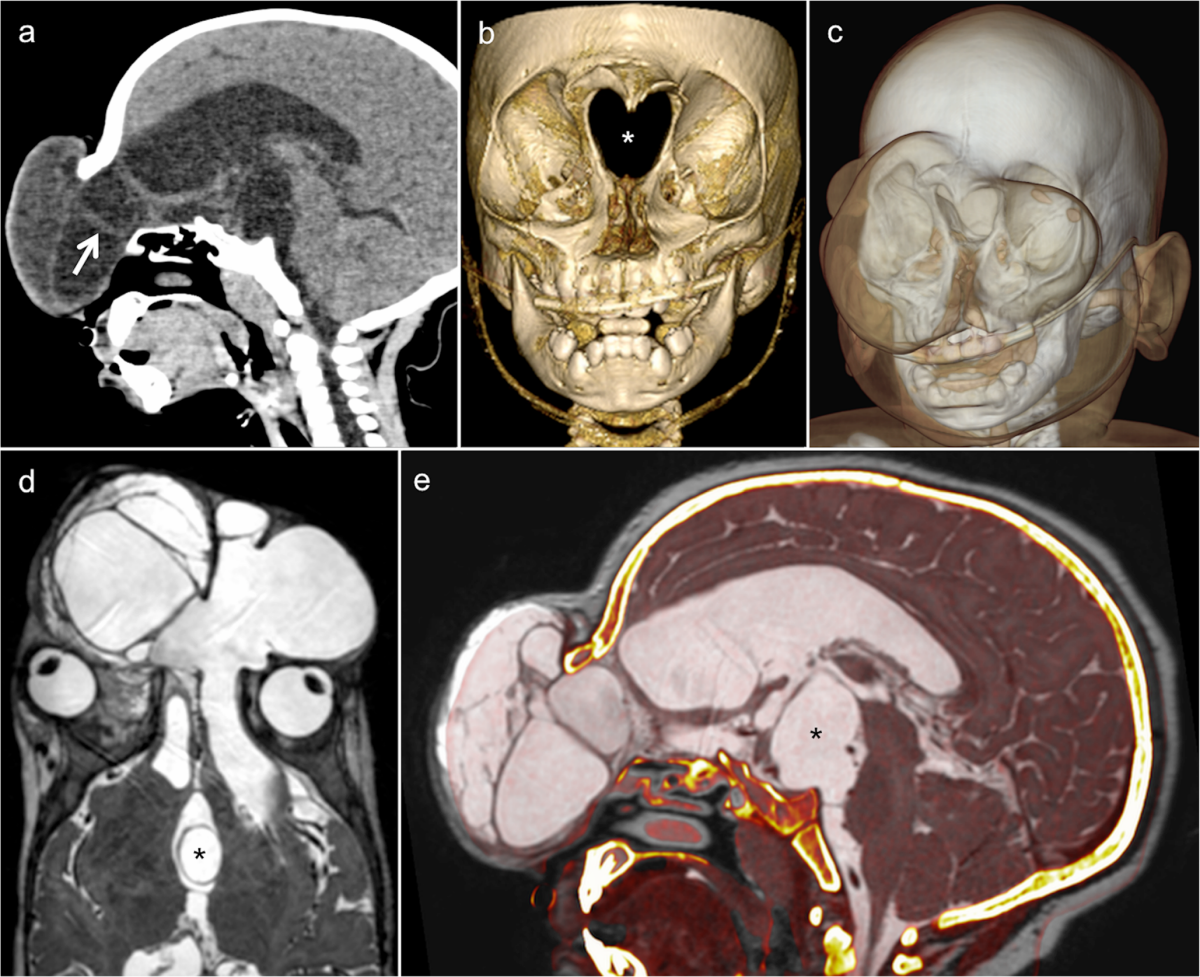
Meningoencephalocele in a 13-month-old boy of African origin with a large midline facial mass. **a** Sagittal CT (brain window) shows herniation of cranial content through a bony defect located in the anterior skull base. The herniation contains a cyst-like mass with multiple septations and lined by glial tissue and/or meninges (*arrow*). **b** The exact size and configuration of the bony defect, as well as the extent of orbital bony malformation, is better appreciated on the three-dimensional CT VR reconstruction of the bony skull (*asterisk*). **c** Three-dimensional CT VR illustrating the extent and position of the meningoencephalocele with respect to the craniofacial skeleton. **d** Axial T2 shows cystic cerebrospinal fluid (CSF)-filled meningocele structures displacing the globes laterally and a suprasellar cyst (*asterisk*). **e** Fused sagittal CT and T2 illustrate CSF, parenchymal and bony abnormalities. Large suprasellar cyst (*asterisk*) lined by Liliequist membrane

Imaging plays a major role for the delineation of parenchymal and bone abnormalities, and helps in determining the surgical approach (Fig. 13). High-resolution CT depicts the bony defect and shows herniation of brain parenchyma and meninges. MRI illustrates the contents of the cephalocele and the associated brain abnormalities. High-resolution three-dimensional T2 sequences, such as constructive interference steady state (CISS), fluid attenuation inversion recovery (FLAIR) and three-dimensional T1 sequences, are useful to depict the degree of parenchymal herniation [69]. In patients with CSF rhinorrhoea and otorrhoea, detection of the site of the CSF leak is challenging and may involve several imaging modalities, including invasive procedures such as CT and MRI cisternography [68]. For the diagnosis of active CSF leak, CT cisternography has a sensitivity of 80–85% [68]. MRI cisternography is a better choice for patients with recurrent or inactive CSF fistulas, with a reported sensitivity as high as 100%. Laboratory studies are helpful by evaluating the content of beta-2 transferrin in nasal secretions [68]. Occasionally, intraorbital cephaloceles can mimic an orbital dermoid or epidermoid cyst. In these situations, cephaloceles present as extraconal masses in the medial orbit without clearly identifiable connection to the intracranial contents. The treatment of cephaloceles involves a multidisciplinary approach and various surgical techniques to treat the cause, symptoms and secondary complications [70].

## Conclusion

This article provides a comprehensive approach to the understanding of the clinical, radiologic and histologic features of masses and mass-like lesions of developmental and genetic origin involving the craniofacial skeleton, with emphasis on the imaging findings that are essential for diagnosis, treatment and surveillance.

## Abbreviations

ADC: Apparent diffusion coefficient
AVM: Arterial vascular malformation
CBCT: Cone beam CT
CEMRI: Contrast-enhanced MRI
CISS: Constructive interference steady state
CNS: Central nervous system
CSF: Cerebrospinal fluid
CT: Computed tomography
DC: Dermoid cyst
DVM: Desmoplastic variant of medulloblastoma
DWI: Diffusion-weighted imaging
FD: Fibrous dysplasia
FDG: F18-Fluoro-deoxy-D-glucose
FLAIR: Fluid attenuation inversion recovery
H-E: Haematoxylin and eosin staining
LM: Lymphatic malformations
MPNST: Malignant peripheral nerve sheath tumour
MRI: Magnetic resonance imaging
NBCCS: Nevoid basal cell carcinoma syndrome
NDSC: Nasal dermoid sinus cyst
NF1: Neurofibromatosis type 1
OKC: Odontogenic keratocyst
OPT: Orthopantomography
PET-CT: Positron emission tomography computed tomography
PNF: Plexiform neurofibroma
TM: Torus mandibularis
TMax: Torus maxillaris
TMJ: Temporomandibular joint
TP: Torus palatinus
US: Ultrasonography
VM: Vascular malformation
VR: Volume rendering
VVM: Venous vascular malformation
